# Prenatal Environment and Neurodevelopmental Disorders

**DOI:** 10.3389/fendo.2022.860110

**Published:** 2022-03-15

**Authors:** Miyuki Doi, Noriyoshi Usui, Shoichi Shimada

**Affiliations:** ^1^ Department of Neuroscience and Cell Biology, Graduate School of Medicine, Osaka University, Suita, Japan; ^2^ United Graduate School of Child Development, Osaka University, Suita, Japan; ^3^ Global Center for Medical Engineering and Informatics, Osaka University, Suita, Japan; ^4^ Addiction Research Unit, Osaka Psychiatric Research Center, Osaka Psychiatric Medical Center, Osaka, Japan

**Keywords:** DOHaD, prenatal environment, preterm birth (PTB), low birth weight (LBW), neurodevelopmental disorders (NDDs), autism spectrum disorder (ASD)

## Abstract

The internal and external environment of the mother during the developmental stages of the fetus affects the offspring’s health. According to the developmental origins of health and disease (DOHaD) theory, environmental factors influence the offspring and also affect health in adulthood. Recently, studies based on this theory have gained attracted attention because of their clinical utility in identifying the risk groups for various diseases. Neurodevelopmental disorders (NDDs) such as autism spectrum disorder (ASD) and attention-deficit hyperactivity disorder (ADHD) can be caused by exposure to certain prenatal environments during pregnancy. This review describes the latest findings on the effect of prenatal environment on the onset mechanism of NDDs based on the DOHaD theory. Unravelling the molecular mechanisms underlying the pathogenesis of NDDs is important, because there are no therapeutic drugs for these disorders. Furthermore, elucidating the relationship between the DOHaD theory and NDDs will contribute to the popularization of preventive medicine.

## Introduction

The DOHaD theory is various environments during development induce predictive adaptive responses that anticipate later environments, and that the degree of adaptation between these environments and later environments is related to future disease risk. For example, overnutrition and undernutrition in early childhood are expected to occur as the predictive adaptive response such as obesity and diabetes in adulthood. Previous epidemiological studies have demonstrated that the prenatal and/or postnatal environment is associated with the risk of various diseases at the later stages of life (1, 2). In the 1980s, an epidemiological study reported by Baker et al. has proposed the fetal programming hypothesis, which states that future health conditions are destined based on the early environment during prenatal and/or postnatal developmental periods (1). The DOHaD theory suggests that the characteristics acquired in early fetal developmental periods due to a certain environment become health risk factors in adulthood (1, 2). Based on the DOHaD theory, it is possible to predict and identify high-risk groups for various diseases, therefore it is gaining attention in the field of preventive medicine, early intervention, and therapeutic treatment.

**Figure 1:**
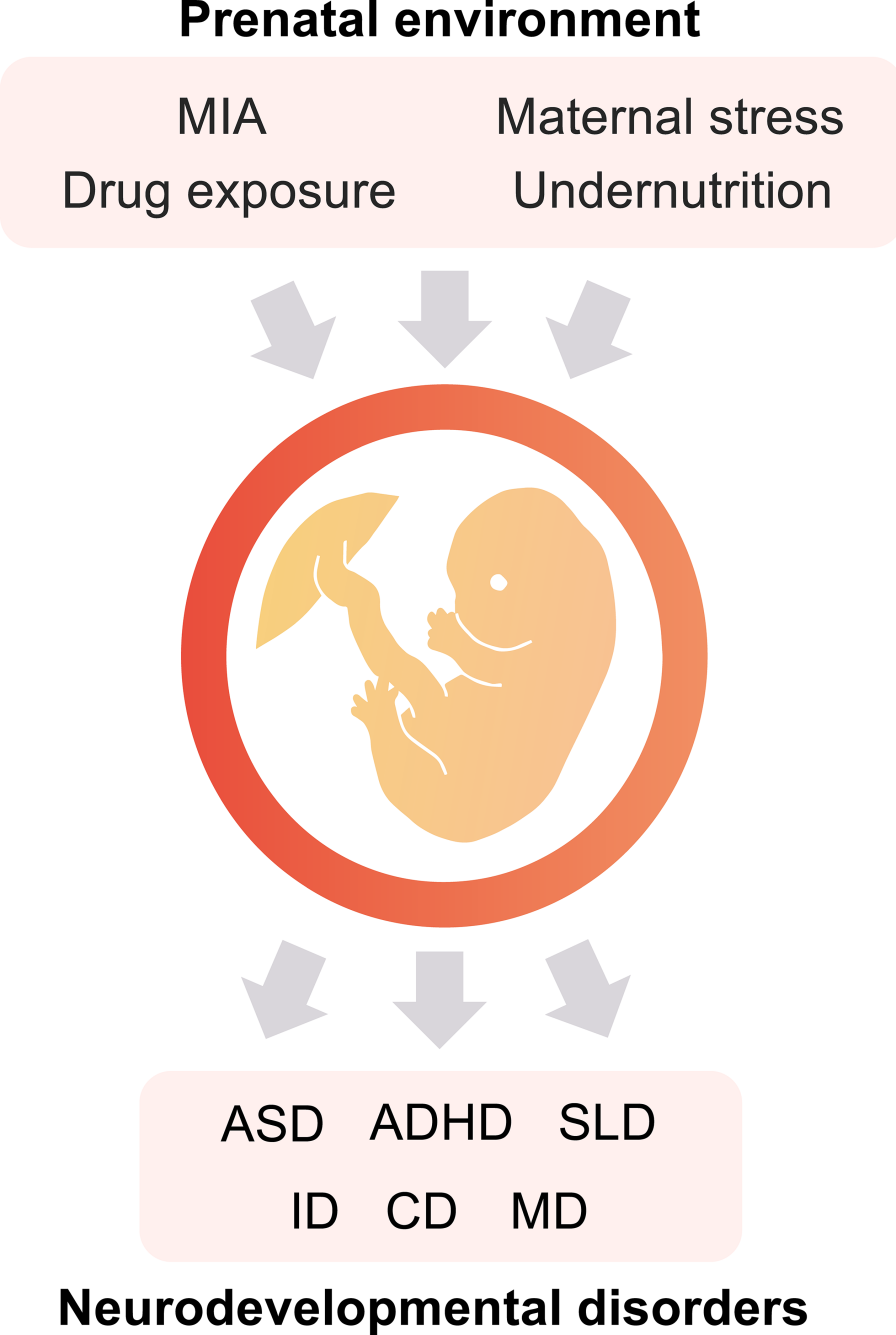
The prenatal environment impacts the offspring’s health. Maternal immune activation (MIA), exposure to specific drugs, maternal stress, and undernutrition during the fetal period are potential risk factors for the onset of neurodevelopmental disorders (NDDs). ASD, autism spectrum disorder; ADHD, attention-deficit hyperactivity disorder; SLD, specific learning disorder; ID, intellectual disabilities; CD, communication disorders; and MD, motor disorders.

In the prenatal environment, maternal immune activation (MIA), stress, undernutrition, and drug exposure are well-known as the environmental factors that affect the future health of the offspring (3–5). These environmental factors are associated with various NDDs (**Figure 1**) and psychiatric disorders such as ASD, ADHD, schizophrenia, and depression (5). ASD is a NDD characterized by social communication deficits, repetitive behaviors, and hyperesthesia/hypesthesia. The prevalence of ASD has been reported as a one in 54 (1.85%) in the US (6). ASD can be caused by both genetic and environmental factors. ADHD is characterized by hyperactivity, attention deficits, and impulsivity. The worldwide prevalence of ADHD is 5.29% (7). ADHD is also caused by genetic and/or environmental factors.

**Figure 2:**
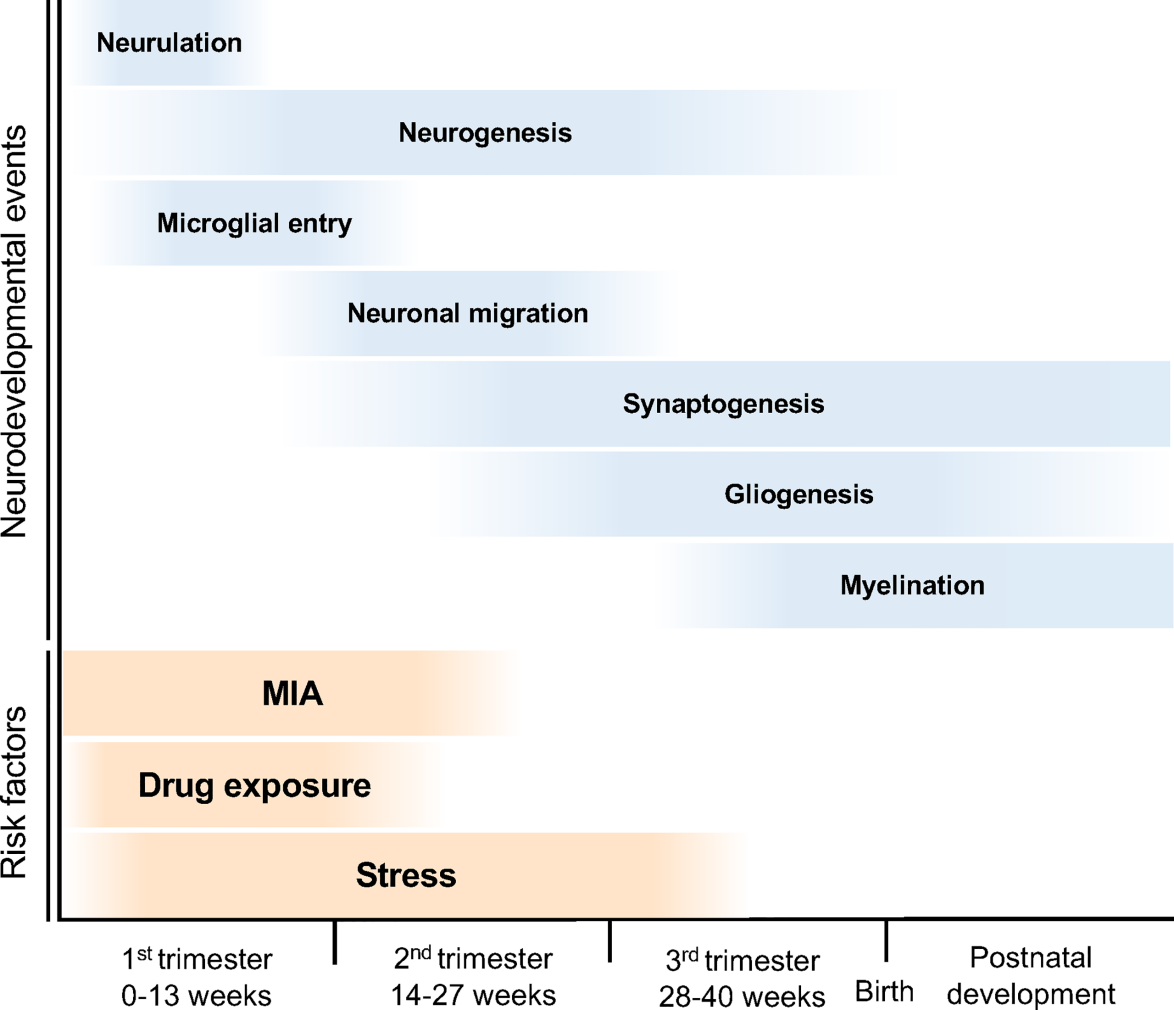
Neurodevelopmental trajectory and risk factors for NDDs. The blue bars indicate neurodevelopmental events during fetal brain development. After neural tube formation in the ectoderm (neurulation), brain vesicles are formed and neuroepithelial cells produce neural stem cell progenitors and neurons (neurogenesis). Neural progenitor cells also produce astrocytes and oligodendrocytes (gliogenesis). The developing neurons migrate, differentiate into specific subtypes, and form synapses and myelin. The orange bars indicate the risk periods for NDDs. MIA during pregnancy can cause NDDs; in particular, the risk of ASD onset increases in the first half of pregnancy. Maternal stress and drug exposure during pregnancy can also cause NDDs. For example, thalidomide and valproic acid exposure are known risk factors for ASD onset.

The developmental stages of the human embryonic brain are divided into three periods (**Figure 2**). In the first trimester (0–13 weeks of gestation), the neural tube is formed in the ectoderm, and the neuroepithelial cells that form the neural tube produce neural progenitor cells and neurons (8). These neurons migrate to the cortical layer and eventually begin to form synapses during the late first to second trimesters (14–27 weeks of gestation) (8). In the third trimester, neuronal axons and glia are produced from the glial progenitor cells such as astrocytes and oligodendrocytes, and are integrated into neural circuits (9). Brain morphology and plasticity continually develop after birth (8). Courchesne et al. reported that ASD associated genes continuously expressed in all trimesters first to third (10). However, the specific period of risk factors such as maternal infection, exposure to certain drug, and stress in the onset of ASD is reported (3, 5, 11) (**Figure 2**). Together, neurodevelopmental abnormalities may occur in the prenatal period (first to third trimesters), when the brain is particularly sensitive and fragile to the surrounding environment, subsequently results in NDDs (10).

To understand the pathophysiology underlying NDDs, identification of causative genes in patients, and research and development using animal models have been promoted. Exploring the environmental factors associated with NDDs and unravelling the underlying mechanisms based on the DOHaD theory is meaningful as it can correlate research to clinical practice and treatments. The present review describes the latest findings on the correlation between the prenatal environment and the onset mechanism of NDDs based on the DOHaD theory.

## Maternal Immune Activation

MIA is an inflammatory response triggered by pathogenic infection and autoimmune diseases in the mother. According to the previous epidemiological studies, MIA is known to be a risk factor for NDDs and psychiatric disorders such as ASD and schizophrenia (12). Toxoplasma, rubella, cytomegalovirus, herpes simplex virus, and Zika virus are vertically transmitted to the fetus, affecting its development and resulting in severe complications such as miscarriage and malformations (5). However, the infections with non-vertically transmitted pathogens such as influenza during pregnancy can cause NDDs in the offspring.

Inflammatory cytokines produced in the mother because of infection directly damage the fetal brain *via* the placenta. In a mouse model of MIA induced by poly(I:C), it was demonstrated that interleukin (IL)-17 produced from T-helper (Th)-17 cells in the mother’s body reach the fetal brain *via* the placenta and induce cell death *via* IL-17 receptors expressed in the fetal brain, resulting in ASD-like behavioral and morphological brain abnormalities (13, 14). In MIA-induced ASD model mice, the decreases of synaptic density and expression levels of synapse formation associated proteins were also reported (15). Such synaptic dysfunction is well known in pathophysiology of ASD (16). For example, mutations of neuroligin encoding genes *NLGN3* and *NLGN4* related with synapse formation have been reported in ASD patients (17). The mutated neuroligin displayed abnormal localization in synapse and resulted in synaptic dysfunction (18). Epidemiological studies have also reported that MIA caused by autoimmune diseases increases the risk of NDDs (19). Recently, it was reported that antioxidant reduce perinatal inflammation in the placenta of the MIA mouse model, suggesting that it may be possible to prevent and treat NDDs by suppressing the inflammation induced by MIA (20). These studies suggest that MIA-induced inflammation and cytokines may impair placental function and lead to disruption of its barrier function, resulting exposures of the fetus to toxic substances and disruptions of nutrient supply.

Individual differences in susceptibility to MIA have been reported to be caused by deficiency of micronutrient (5, 21, 22). A sufficient supply of essential elements such as of minerals and vitamins is essential for typical fetal development (5). It is well known that folic acid deficiency in pregnant women causes neural tube defects (23). Harvey et al. also reported that iron deficiency during pregnancy enhances cytokine induction by MIA (24). Moreover, vitamin D, zinc, and omega fatty acids are essential factors for typical fetal development (21, 22, 25). For example, dyslipidemia has been reported in children with ASD (26).

Stress during pregnancy is also known to induce brain inflammation and influence the fetal brain development (27). Epidemiological studies have reported that stress during pregnancy increases the risk of ASD and ADHD (3, 28). It is well known that increased blood levels of adrenocorticotropic stress-related hormones such as cortisol and corticosterone are a response to stress. In primates, cortisol, which has a strong physiological activity, is converted into its inactive form by Hydroxysteroid 11-β dehydrogenase 2 (HSD11B2) enzyme. Blood cortisol during pregnancy is converted into inactivated forms in the placenta to prevent fetal exposure to cortisol (29). However, HSD11B2 expression is downregulated in the placentas of chronic stressed mothers (30). Therefore, fetuses are exposed to high concentrations of cortisol, resulting in developmental delays and NDDs (28, 30). In rodents, exposure to high concentrations of stress-related hormone in early life stages decreases the expression of *Hsd11b1*, which encodes a stress-related hormone activity regulator enzyme in the neocortex (31).

## Drug Exposure

Disturbance of the prenatal environment due to drug exposure also causes NDDs (4, 11, 32). In the 1960s, thalidomide was used for treating morning sickness in pregnant women; however, it resulted in fetal malformations. In 2004, Miller et al. reported that exposure to thalidomide during early pregnancy causes ASD in the offspring (11). However, the detailed mechanism by which thalidomide causes ASD remains unknown. Recently, Ando et al. reported that exposure to thalidomide caused brain shrinkage in zebrafish due to complex formation of thalidomide with E3 ubiquitin ligase Cereblone (CRBN), which negatively regulates neural stem cell proliferation (33). Miller et al. reported that CRBN degrades Zbtb16 and Sall4 resulting in limb teratogenicity in chick embryos (34). *ZBTB16* is a transcription factor essential for cell proliferation and differentiation (35). In *Zbtb16* knockout (KO) mice, it has been reported that Zbtb16 regulates neural stem cell proliferation during the early embryonic stage (36). Recent genome-wide association studies (GWASs) have identified the mutations in *ZBTB16* in patients with ASD (37, 38). It has been demonstrated that *Zbtb16* KO mice show ASD-like behaviors such as social impairment and repetitive behaviors (39). Together, these studies suggest that Zbtb16 dysfunction due to activation of CRBN by thalidomide exposure during pregnancy results in impairments of neural proliferation and differentiation which alternatively lead to NDDs such as ASD.

Exposure to valproic acid (VPA), an antiepileptic drug, also affects neural development (4, 32, 40). Previous epidemiological studies have shown that the VPA administration during pregnancy, particularly during fetal brain development increases the risk of ASD onset (4, 41). Furthermore, the study demonstrated that VPA administration at mouse embryonic day 12.5 (E12.5) decreases the number of neurons in the prefrontal and somatosensory cortexes (32). In addition, Kataoka et al. reported that VPA administration induced hyperacetylation of genomic DNA and behavioral abnormalities including ASD-like features (32).

A relationship between pregnant women taking antidepressants such as selective serotonin reuptake inhibitors (SSRIs) and the onset of ASD has been suggested. Serotonin plays a role in fetal and postnatal brain development (42). Mutations in *SLC6A4* which encodes serotonin transporter that regulates serotonin levels in the synaptic cleft has been reported in patients with ASD (43, 44). Disturbance of the serotonergic system is involved in the pathophysiology of ASD (45). Serotonin levels in the brains of ASD patients have been reported to be lower than those in individuals with typical developments (45). Furthermore, a decrease in the density of serotonin transporter (SERT) in ASD patients has also been reported (46). Thus, it is possible that SSRIs in pregnant women cause disturbances in serotonin levels. However, previous studies have reported that the underlying mechanism of the association between the use of SSRIs by pregnant women and the onset of ASD is unclear (47, 48).

Additionally, epidemiological studies have demonstrated that exposure to other agents such as organophosphates and chemicals produced by smoking increases the risk of ASD, ADHD, and cognitive impairments in the offspring (49, 50). Prenatal cocaine exposure also affects offspring’s social behaviors such as attachment and play behaviors (51). Prenatal cannabis exposure increases the incident risks of ASD, intellectual disability, and learning disorders in the offspring (52). It is also known that prenatal exposure to alcohol induces fetal brain development and fetal alcohol spectrum disorder including behavioral difficulties (53). Therefore, exposure to drugs, alcohol, toxic substances, and psychostimulants during pregnancy should be treated with caution because of the high risks of NDDs.

## Preterm Birth and Low Birth Weight

Preterm birth and low birth weight can also lead to ASD and ADHD. Maternal stress, undernutrition, and infection during pregnancy can induce abnormal births (54, 55). In 2013, Singh et al. examined the mental health condition of premature offspring with less than 37 weeks of gestation and low birth weight of less than 2500g, and found an increases in the onset probability of NDDs in these offspring (56). Pacheco proposed a relationship between the climate changes and maternal and child health. Climate changes such as storms, heat, floods, foods insecurity, and air pollution adversely lead to maternal stress, food insecurity, undernutrition, and toxic exposure, eventually leading to the onset of preterm birth, low birth weight, and NDDs (55).

Generally, intrauterine growth restriction (IUGR) results in low birth weight. Baschat reported IUGR is induced not only by undernutrition but also by reduced placental blood flow and hypoxemia (57). Functional insufficiency of the placenta such as dysfunction of the glucocorticoid barrier (see *Maternal Immune Activation* section) is also linked to the onset of IUGR (30). In the IUGR offspring, the brain volume in the gray matter of the limbic region is reduced; however the regional volumes of the frontoinsular, frontal, and temporal-parietal areas are expanded (58). These studies suggest that the abnormal brain development caused by preterm birth, low birth weight, and IUGR are risk factors for NDDs.

## Discussion

This review entailed the risk factors for NDDs based on the DOHaD theory. The DOHaD theory proposed a relationship between fetal development and its adult health. The underlying phenomenon for this correlation can be explained using epigenetics. Epigenetics is a molecular mechanism that alters gene expression by chemical modification on DNA without affecting the genomic sequences. It is influenced by external environments, and thus it reflects the interaction of genetic and environmental factors. Previous reports show that there are age-associated DNA methylation changes in paternal sperm (59). Besides epigenetics, *de novo* mutations that can accumulate in aged father’s sperm have also been reported to be at risk for NDDs (60). In terms of the relationship between parental age and ASD onset, it has been reported that the aging of the father’s sperm affects ASD onset more strongly than maternal age (61). The prenatal environment in fetal developmental stages affects the onsets of various diseases such as NDDs, psychiatric disorders, metabolic disorders, and high blood pressure. In conclusion, according to the DOHaD theory, understanding the mechanisms underlying the prenatal environment and fetal development can promote preventive medicine, early intervention, and therapeutic treatment based on individual risks.

## Author Contributions

MD: Writing - Original Draft, Visualization. NU: Conceptualization, Writing - Original Draft, Writing - Review and Editing, Project administration, Funding acquisition. SS: Writing - Review and Editing, Supervision, Funding acquisition. All authors contributed to the article and approved the submitted version.

## Funding

This work was supported by the Japan Society for the Japan Science and Technology Agency (JST) Center of Innovation Program (COI Program) (JPMJCE1310) to NU and SS; the Promotion of Science (JSPS) Grant-in-Aid for Scientific Research (C) (20K06872) to NU; JSPS Grant-in-Aid for Early-Career Scientists (18K14814) to NU; JSPS Grant-in-Aid for Challenging Research (20K21654) to NU and SS; Uehara Memorial Foundation to NU; Takeda Science Foundation to NU; SENSHIN Medical Research Foundation to NU; Osaka Medical Research Foundation for Intractable Diseases to NU; Public Health Science Foundation to NU; Eli Lilly Japan Research Grant to NU.

## Conflict of Interest

The authors declare that the research was conducted in the absence of any commercial or financial relationships that could be construed as a potential conflict of interest.

## Publisher’s Note

All claims expressed in this article are solely those of the authors and do not necessarily represent those of their affiliated organizations, or those of the publisher, the editors and the reviewers. Any product that may be evaluated in this article, or claim that may be made by its manufacturer, is not guaranteed or endorsed by the publisher.
